# Prognostic Value of CT-Attenuation and ^18^F-Fluorodeoxyglucose Uptake of Periprostatic Adipose Tissue in Patients with Prostate Cancer

**DOI:** 10.3390/jpm10040185

**Published:** 2020-10-22

**Authors:** Jeong Won Lee, Youn Soo Jeon, Ki Hong Kim, Hee Jo Yang, Chang Ho Lee, Sang Mi Lee

**Affiliations:** 1 Department of Nuclear Medicine, Catholic Kwandong University College of Medicine, International St. Mary’s Hospital, 25, Simgok-ro 100 beon-gil, Seo-gu, Incheon 22711, Korea; sads00@naver.com; 2 Department of Urology, Soonchunhyang University Cheonan Hospital, 31, Suncheonhyang 6-gil, Dongnam-gu, Cheonan, Chungcheongnam-do 31151, Korea; ysurol@schmc.ac.kr (Y.S.J.); urokim@schmc.ac.kr (K.H.K.); c78154@schmc.ac.kr (H.J.Y.); leech@schmc.ac.kr (C.H.L.); 3 Department of Nuclear Medicine, Soonchunhyang University Cheonan Hospital, 31 Suncheonhyang 6-gil, Dongnam-gu, Cheonan, Chungcheongnam-do 31151, Korea

**Keywords:** fluorodeoxyglucose F18, periprostatic adipose tissue, positron emission tomography, prognosis, prostate cancer

## Abstract

This study aimed to assess the prognostic value of computed tomography (CT)-attenuation and ^18^F-fluorodeoxyglucose (FDG) uptake of periprostatic adipose tissue (PPAT) for predicting disease progression-free survival (DPFS) in patients with prostate cancer. Seventy-seven patients with prostate cancer who underwent staging FDG positron emission tomography (PET)/CT were retrospectively reviewed. CT-attenuation (HU) and FDG uptake (SUV) of PPAT were measured from the PET/CT images. The relationships between these PPAT parameters and clinical factors were assessed, and a Cox proportional hazard regression test was performed to evaluate the prognostic significance of PPAT HU and SUV. PPAT HU and SUV showed significant positive correlations with tumor stage and serum prostate-specific antigen level (PSA) (*p* < 0.05). Patients with high PPAT HU and SUV had significantly worse DPFS than those with low PPAT HU and SUV (*p* < 0.05). In multivariate analysis, PPAT SUV was a significant predictor of DPFS after adjusting for tumor stage, serum PSA, and tumor SUV (*p* = 0.003; hazard ratio, 1.50; 95% confidence interval, 1.15–1.96). CT-attenuation and FDG uptake of PPAT showed significant association with disease progression in patients with prostate cancer. These imaging findings may be evidence of the role of PPAT in prostate cancer progression.

## 1. Introduction

Prostate cancer is the second most commonly diagnosed cancer and fifth leading cause of cancer death in men worldwide [1]. Obese patients show a tendency for higher Gleason grade prostate cancer and increased risk of treatment failure and mortality, and it is now recognized that the characteristics as well as clinical outcomes of prostate cancer are significantly associated with obesity [2,3]. However, other studies failed to show a significant relationship with clinical outcomes of prostate cancer [4,5]. This discrepancy may be due to the fact that body mass index (BMI), which is used to define obesity, provides no insight into the amount of adipose tissue [6,7,8]. Furthermore, adipose tissue characteristics are known to be differ according to location of adipose tissue, suggesting the significance of its distribution [9,10] Therefore, researchers have tried to directly measure the quantities of specific adipose tissue depots, such as subcutaneous (SAT) and visceral (VAT) adipose tissue, using computed tomography (CT) and magnetic resonance imaging (MRI) and evaluate their clinical implications in prostate cancer patients [11,12,13].

Recently, several studies have focused on the role of periprostatic adipose tissue (PPAT) in the development and progression of prostate cancer. PPAT is generally defined as adipose tissue that surrounds the prostate gland [10,14]. PPAT is often considered as a part of VAT, but it is not located within the peritoneal cavity and has different biological characteristics from VAT and SAT [10,14,15]. In vitro studies revealed that PPAT could lead to the development of prostate cancer, and crosstalk between tumor cells and PPAT cells was found to significantly contribute to the proliferation, progression, and dissemination of prostate cancer [10,14,16,17,18,19]. In clinical imaging studies, the quantity of PPAT has been used to estimate the characteristics of PPAT. However, results have been inconsistent with regard to the association between quantitative PPAT parameters and aggressiveness of prostate cancer [8,11,13,20,21]. 

In addition to the quantity of adipose tissue, recent studies have employed CT-attenuation on non-contrast enhanced CT images and ^18^F-fluorodeoxyglucose (FDG) uptake on positron emission tomography (PET) to investigate the clinical value of qualitative characteristics of adipose tissue in cancer patients [22,23]. These two imaging parameters were found to be related to fibrotic and inflammatory changes in adipose tissue and were significantly associated with the prognosis of several malignant diseases [22,23,24,25]. Considering the significant in vitro interaction between PPAT and prostate cancer cells [10,14,17], qualitative characteristics of PPAT on imaging studies may be associated with clinical characteristics and progression of prostate cancer. However, until now, no studies have evaluated the clinical implication of qualitative PPAT imaging parameters in patients with prostate cancer.

Therefore, we measured the CT-attenuation and FDG uptake of PPAT on staging FDG PET/CT images and investigated their relationship to disease progression-free survival (DPFS) in prostate cancer patients. 

## 2. Materials and Methods

### 2.1. Study Population

Clinical and radiological data of patients with histopathologically confirmed prostate cancer who underwent staging FDG PET/CT with no prior treatment between January 2013 and December 2017 at our medical center were retrospectively reviewed. Of these patients, we excluded patients (1) who received only supportive care with no curative or palliative treatment after the staging examinations; (2) who were lost to follow-up within 12 months after the initial treatment without events; (3) who had history of another malignant disease; and (4) who had PET/CT images that were inappropriate for measuring SAT, VAT, and PPAT parameters due to previous abdominal or orthopedic surgery. After exclusion, a total of 77 patients were finally included in the study. 

For prostate cancer staging, all patients initially underwent physical examination, serum prostate-specific antigen (PSA) measurement, transrectal ultrasonography, contrast-enhanced CT of the abdomen and pelvis, MRI of the prostate gland, and bone scintigraphy. They subsequently underwent staging FDG PET/CT to investigate suspicious metastatic lesions seen on other imaging examinations or relatively high serum PSA level in consideration of tumor stage. Based on the results of staging examinations and the patients’ clinical condition, curative or palliative treatment, including surgical resection, radiotherapy, and hormone therapy, was administered. After the initial treatment, all patients were routinely followed up at intervals of 3–6 months. 

This retrospective observational study was conducted in accordance with the Declaration of Helsinki as revised in 2013, and the protocol was approved by the Institutional Review Board of Soonchunhyang University (Ethic code: 2020-08-052, date: 5 August 2020). Because of its retrospective nature, the requirement to obtain informed consent was waived by the board.

### 2.2. FDG PET/CT

FDG PET/CT was performed from the skull base to the proximal thigh in the supine position using a dedicated PET/CT scanner (Biograph mCT 128, Siemens Healthineers, Knoxville, TN, USA) in all enrolled patients. Before PET/CT scanning, patients were instructed to fast for >6 h and 4.07 MBq/kg of FDG was intravenously injected after confirmation of blood glucose level <200 mg/dL. One hour after FDG administration, a noncontrast-enhanced CT scan for attenuation correction was performed at 100 mA and 120 kVp with a slice thickness of 5 mm, followed by PET scan at 1.5 min per bed position. PET images were reconstructed with a point-spread-function-based Gauss and Allpass filter algorithm and time-of-flight reconstruction with attenuation correction.

### 2.3. Image Analysis

**Figure 1:**
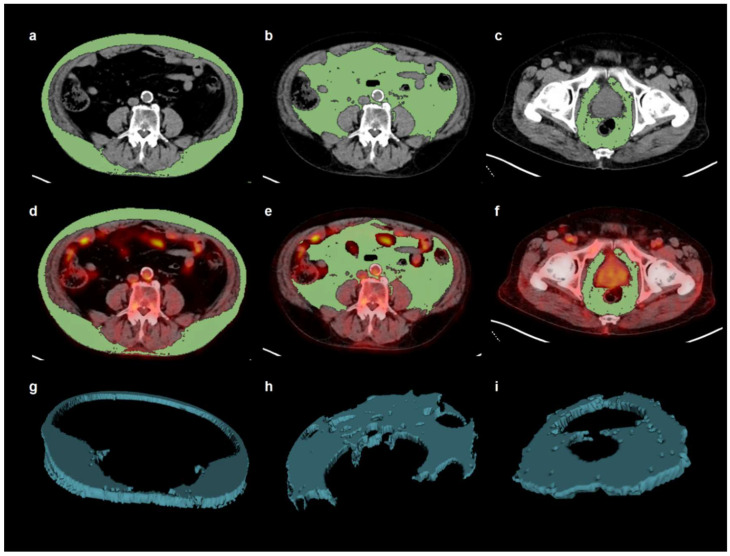
Example of measurement of computed tomography (CT)-attenuation and standardized uptake value (SUV) of subcutaneous adipose tissue (SAT), visceral adipose tissue (VAT), and periprostatic adipose tissue (PPAT). Using a CT-attenuation range between −200 and −50 Hounsfield units, the area of SAT (**a**) and VAT (**b**) were automatically delineated on three consecutive transaxial images at the level of the L4-L5 spine, and the area of PPAT (**c**) at the level of the pubic symphysis. The areas of SAT, VAT, and PPAT on the CT images were exported to the corresponding fused PET/CT images. Physiologic FDG activity in the vessels, urine, and bowel, which could affect the measurement of SUV of adipose tissue were removed from the areas of SAT (**d**), VAT (**e**), and PPAT (**f**) on the fused PET/CT images. Afterwards, the three-dimensional structures of the SAT (**g**), VAT (**h**), and PPAT (**i**) were automatically created for each area on CT and PET/CT images. Mean CT-attenuation was calculated from the three-dimensional structures on CT areas and the mean SUV was measured using the three-dimensional structures on the fused PET/CT.

Two nuclear medicine physicians retrospectively reviewed PET/CT images and measured the PET/CT parameters of the primary tumor and adipose tissue while blinded to the patients’ clinical information. For the primary tumor, a spheroid-shaped volume-of-interest (VOI) was drawn to include the entire lesion, and the maximum SUV of the primary tumor was measured. For prostate cancer lesions with no discernibly increased FDG uptake, the VOI was drawn according to tumor location on CT and MRI images. For adipose tissue, CT-attenuation, expressed as Hounsfield unit (HU), and the mean standardized uptake value (SUV) of SAT, VAT, and PPAT were measured using an United Stated Food and Drug Administration-approved medical image viewer (OsiriX MD 11.0.3, Pixmeo, Geneva, Switzerland) as previously described [22,23,26]. Three consecutive CT images were selected at the level of the L4-L5 spine for measuring SAT and VAT parameters, and at the level of the pubic symphysis for measuring PPAT parameters (Figure 1) [11,27]. PPAT was defined as the area at the back side of the pubic bones, along the lateral border of the obturator internus muscle, and the anterior side of the gluteus maximum muscle and coccygeal bone [8,11]. A threshold of −50 to −200 HU was used for identifying the adipose tissue area on CT images. The SAT, VAT, and PPAT areas on those three CT images were automatically selected. Accordingly, their three-dimensional structures of SAT, VAT, and PPAT were automatically created and their mean HU was computed. Afterwards, the areas of SAT, VAT, and PPAT on the CT images were exported to the corresponding PET images. After removing physiologic FDG activity in the vessels, urine, and bowel, which could affect the measurement of the SUV of adipose tissue, the mean SUV of the SAT, VAT, and PPAT were calculated.

### 2.4. Statistical Analysis

Based on the height and weight measured at the time of FDG PET/CT, BMI was calculated and all patients were classified as underweight/normal weight (<23.0 kg/m^2^) or overweight/obese (≥23.0 kg/m^2^) according to the recommendation for Asian populations [28]. To evaluate differences in CT-attenuation and FDG uptake between SAT, VAT, and PPAT, one-way repeated measures analysis of variance and pairwise multiple comparisons with the Bonferroni correction were performed. The Kruskal-Wallis and Mann-Whitney U tests were performed to assess differences in adipose tissue parameters according to Gleason grade and tumor stage. Spearman rank correlation coefficients were calculated to investigate the relationship between continuous variables. For survival analysis, the prognostic significance of PPAT HU and SUV, as well as other clinical factors, in predicting DPFS was investigated using univariate and multivariate Cox proportional hazards regression. Survival time was defined as time from the initial treatment to detection of disease progression. Patients who showed no disease progression on follow-up imaging studies were censored at the day of the last follow-up visit. For the multivariate survival analysis of the association between PPAT HU and SUV and DPFS, we created 5 different models with 8 covariates. The optimal cut-off values of PPAT HU and SUV were determined using the maximal chi-square method, and accordingly, the Kaplan–Meier method was used to estimate survival curves. Disease progression in each group was compared using Fisher’s exact test. All statistical analyses were performed using R software (version 3.5.3; the R foundation for Statistical Computing, Vienna, Austria) and MedCalc Statistical Software (version 19.4.0; MedCalc Software bvba. Ostend, Belgium). Statistical significance was set at a *p*-value of less than 0.05.

## 3. Results

### 3.1. Baseline Characteristics

The patient characteristics are summarized in Table 1. Of the enrolled patients, 47 (61.0%) were overweight/obese and 33 (42.9%) had serum PSA levels > 20.0 ng/mL. On staging imaging studies, distant metastatic lesions were found in 22 patients (28.6%), and 36 (46.8%) were diagnosed with stage IV prostate cancer. The interval between FDG PET/CT and initial treatment was within two weeks in all patients. The median follow-up duration after the initial treatment was 27.7 months (range, 1.3–83.3 months). At the time of analysis, 21 patients (27.3%) experienced disease progression.

### 3.2. PPAT Parameters

**Figure 2:**
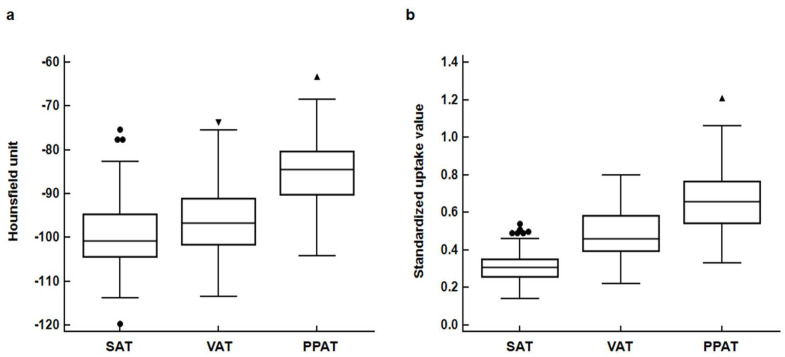
Distribution of computed tomography-attenuation (Hounsfield unit) (**a**) and standardized uptake value (**b**) for subcutaneous adipose tissue (SAT), visceral adipose tissue (VAT) and periprostatic adipose tissue (PPAT). (Central box, the values from the 25 percentile to 75 percentile; middle line in the box, median value; error bar, extending from the minimum value to the maximum value except outside values; circle, an outside value for SAT parameters which is smaller than 25 percentile minus 1.5 times the interquartile range or larger than the 75 percentile value plus 1.5 times the interquartile range; inverted triangle, an outside value for VAT parameters which is larger than the 75 percentile value plus 1.5 tines the interquartile range; positive triangle, an outside value for PPAT parameters which is larger than the 75 percentile value plus 1.5 times the interquartile range)

Comparison of CT-attenuation and FDG uptake according to adipose tissue site showed significant differences in both HU and SUV between SAT, VAT, and PPAT (*p* < 0.001; Figure 2). In pairwise comparisons after Bonferroni correction, the HU and SUV of SAT, VAT, and PPAT showed significant differences (*p* < 0.001), with the highest HU and SUV values observed in PPAT. 

**Figure 3:**
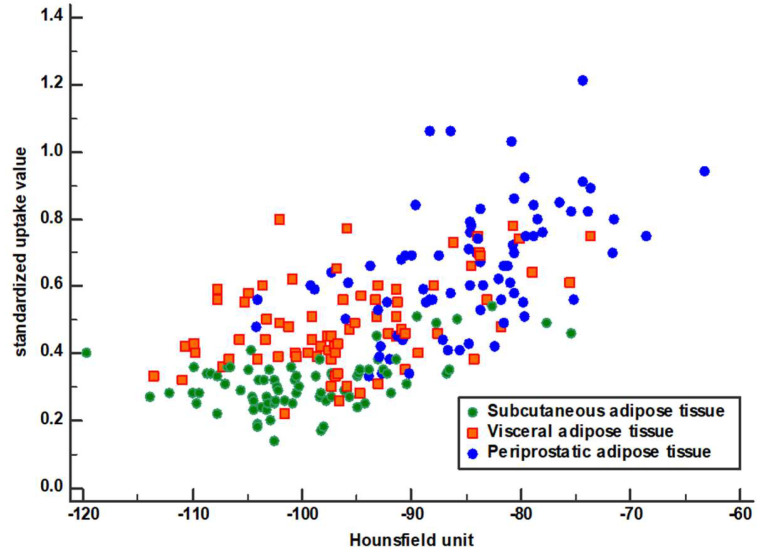
A scatter plot showing the relationship between CT-attenuation and standardized uptake value in subcutaneous, visceral, and periprostatic adipose tissue.

In the assessment of the relationship between CT-attenuation and FDG uptake of adipose tissue, the HU and SUV of PPAT showed a significant moderate positive correlation with each other (*p* < 0.001, r = 0.571; Figure 3). Likewise, HU and SUV of SAT (*p* = 0.00, r = 0.361) and VAT (*p* < 0.001, r = 0.403) also showed significant positive correlations. However, the correlations were weak. 

On correlations of the PPAT parameters with clinical factors, the PPAT parameters showed no significant correlations with BMI (*p* = 0.101 for HU and *p* = 0.161 for SUV) or Gleason grade (*p* = 0.173 for HU and *p* = 0.622 for SUV; Table 2). In terms of tumor stage, patients with T4 stage, N1 stage, and M1 stage showed significantly higher PPAT HU and SUV than those with T2-T3 stage, N0 stage, M0 stage, respectively (*p* < 0.05; Table 2; Supplementary Figure S1). However, all SAT and VAT parameters failed to show a correlation with T stage, and only SAT SUV and VAT HU showed significant positive correlations with N stage and M stage, respectively (Supplementary Table S1). In correlation analysis with serum PSA level and tumor SUV, weak positive correlations were observed between PPAT SUV and serum PSA level (*p* = 0.048, r = 0.216) and tumor SUV (*p* = 0.013, r = 0.282), while PPAT HU showed a significant positive correlation with serum PSA level only (*p* = 0.002, r = 0.411).

### 3.3. Survival Analysis

The influence of adipose tissue imaging parameters and other clinical factors on DPFS was assessed using univariate Cox regression analysis (Table 3). In univariate analysis, both PPAT HU and SUV showed a significant positive association with DPFS (*p* < 0.05). In addition, SAT SUV and VAT SUV were significantly associated with DPFS (*p* < 0.05), whereas SAT HU and VAT HU were not (*p* > 0.05). Among the clinical factors, Gleason grade, serum PSA level, T stage, N stage, M stage, TNM stage, and tumor SUV were significantly associated with DPFS (*p* < 0.05).

The prognostic significance of PPAT HU and SUV was further assessed after adjustment for other covariates included in the univariate survival analysis. Given the number of events as compared with the number of variables included in the multivariate analysis [29], five different multivariate models with eight covariates (age, body mass index, Gleason grade group, TNM stage, serum PSA, tumor SUV, SAT SUV, and VAT SUV) were developed. Because PPAT and SUV showed significant association with each other, they were assessed in a separate model (Table 4). In models with age and BMI (model 1), with Gleason grade and TNM stage (model 2), and with serum PSA and tumor SUV (model 3), both PPAT HU and SUV showed a significant association with DPFS (*p* < 0.05). In a model with adipose tissue parameters (model 4), only PPAT SUV was a significant predictor (*p* = 0.005). Additionally, we created a final multivariate model (model 5) that included four covariates (TNM stage, serum PSA, tumor SUV, and SAT SUV) showing statistical significance in the model 1–4. The results showed that PPAT SUV remained a significant predictor of DPFS (*p* = 0.003; hazard ratio = 1.50 for 0.10 increase; 95% confidence interval 1.15–1.96) as did the with TNM stage (*p* = 0.013), while both serum PSA (*p* = 0.050) and tumor SUV (*p* = 0.054) revealed borderline statistical significance. PPAT HU failed to show a significant association with DPFS in the final model (*p* = 0.343).

**Figure 4:**
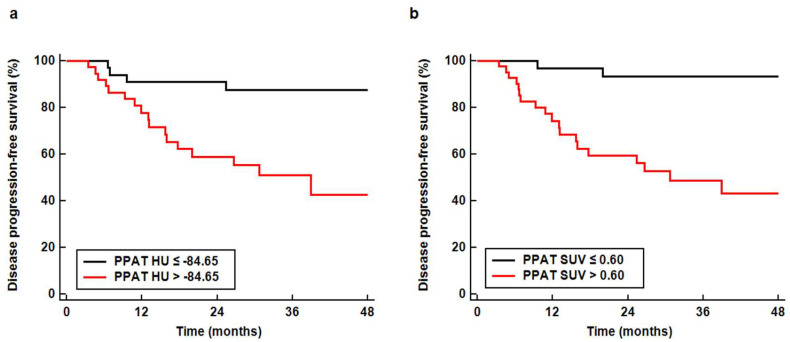
Disease progression-free survival curves according to the computed tomography-attenuation of periprostatic adipose tissue (PPAT HU) (**a**) and standardized uptake value of periprostatic adipose tissue (PPAT SUV) (**b**).

For Kaplan–Meier analysis, the enrolled patients were dichotomized according to the optimal cut-off values of PPAT HU and SUV (−84.65 HU and 0.60, respectively) determined by the maximal chi-square method. The results revealed that patients with high PPAT HU and SUV showed significantly worse DPFS than those with low PPAT HU and SUV (*p* < 0.001 for all; Figure 4). Patients with PPAT HU > −84.65 and PPAT SUV > 0.60 had two-year DPFS rates of less than 60.0% (59.0% for HU and 59.5% for SUV), while those with PPAT HU ≤ −84.65 and PPAT SUV ≤ 0.60 showed two-year DPFS of more than 90.0% (91.0% for HU and 93.3% for SUV). Furthermore, among 45 patients with PPAT SUV > 0.60, 10 (22.2%) had disease progression within one year, compared to only one (3.1%) who experienced disease progression within one year among the 32 with PPAT SUV ≤ 0.60 (*p* = 0.021).

## 4. Discussion

In the present study, both CT-attenuation and FDG uptake of PPAT measured using FDG PET/CT showed significant positive correlations with tumor stage and serum PSA level. In addition, patients with high values of both imaging parameters had a worse prognosis. Moreover, FDG uptake of PPAT was determined to be an independent predictor of DPFS after adjusting for tumor stage, serum PSA, tumor SUV, and SAT SUV in patients with prostate cancer. 

Prostate cancer cells are involved in deleterious bidirectional crosstalk with PPAT adipocytes [10,14,30]. Cancer cells induce substantial phenotypic and functional alterations to adipocytes in the vicinity of them [10,31,32]. They can modify the secretory profile of adipocytes, enhancing the secretion of multiple adipokines, cytokines, and chemokines, such as osteopontin, tumor necrosis factor alpha, interleukin-6, monocyte chemotactic protein-1, and metalloprotease 2, and reducing the secretion of protective adipokines, which can conversely lead to development, proliferation, progression, and migration of cancer cells [14,17,18,31,32,33,34]. Furthermore, these cancer-associated adipocytes also induce inflammatory and fibrotic changes in the adipose tissue and provide an energy source for cancer cells in the form of fatty acids [16,32,35]. In terms of phenotype, these modified adipocytes show dedifferentiated features such as decreased cell size and less intracellular lipid content with altered fatty acid composition, and exhibit fibroblast-like morphology, which are now so-called cancer-associated adipocytes [10,32,36]. This bidirectional crosstalk can create a vicious circle that leads to prostate cancer progression [10,14,30]. In previous studies, PPAT in aggressive prostate cancer had distinct gene expression and transcriptional signatures with high levels of inflammation as compared with PPAT in less aggressive prostate cancer, supporting the significant interactions between cancer cells and PPAT cells [19,37,38,39]. 

Currently, clinical studies have investigated the use of CT-attenuation and FDG uptake of adipose tissue to estimate the qualitative change in adipose tissue in various malignant diseases [22,23,24,25,26,40]. Adipose tissue with increased CT-attenuation on non-contrast enhanced CT was found to show increased extracellular matrix fibrosis and small adipocytes with low lipid content on histopathological evaluation, that are also observed in the adipose tissue with cancer-associated adipocytes [10,23,25,32]. FDG uptake of adipose tissue on PET/CT is thought to be related to the glucose metabolism of adipocytes and inflammatory cells recruited to the adipose tissue, thereby reflecting the degree of inflammatory response in the adipose tissue [22,23,41]. In this study, the CT-attenuation and FDG uptake of PPAT were significantly higher than those of SAT and VAT. This suggests that PPAT exhibits more prominent inflammatory and fibrotic changes than SAT and VAT in prostate cancer patients, which might be caused by the aforementioned bidirectional crosstalk between prostate cancer cells and PPAT [10,14,15]. Moreover, CT attenuation and FDG uptake of PPAT showed a positive moderate correlation, which is closer than those of SAT and VAT, indicating that the qualitative changes in PPAT could lead to changes in both CT-attenuation and FDG uptake. 

In previous studies, the clinical significance of CT attenuation and FDG uptake of SAT and VAT has been reported in cancers that grow in an adipose tissue dominant environment, such as colorectal cancer, gastric cancer, and pancreatic cancer [22,23,41,42]. Increased CT-attenuation and FDG uptake of SAT and VAT were observed in patients with advanced stage disease and aggressive features, and VAT HU and SUV were independent predictors of clinical outcomes, suggesting a significant association between the qualitative features of adipose tissue and cancer progression [22,23,41,42]. Furthermore, in patients with breast cancer, CT attenuation of tumor-adjacent breast adipose tissue was significantly higher than that of the contralateral breast tissue, and was significantly associated with T stage and recurrence risk after curative resection [35]. Since both breast and prostate cancer cells are surrounded by adipose tissue with very close localization with adipocytes [32], it would be reasonable to assume that PPAT qualitative features could also influence the clinical characteristics of prostate cancer. In accordance with our hypothesis, we demonstrated that both PPAT HU and SUV were elevated among patients with advanced stage disease and were associated with worse survival, indicating the significant correlation between qualitative features of PPAT and prostate cancer aggressiveness. Although CT-attenuation and FDG uptake reflect different aspects of the qualitative changes of adipose tissue, considering the significant positive correlation between PPAT HU and SUV, it is not surprising that both PPAT imaging parameters were significantly associated with tumor stage and survival in our study. In contrast, Gleason grade group, which is used to estimate the prostate cancer aggressiveness, showed no significant relationship with either of PPAT imaging parameters. However, because of the small proportion of patients with low Gleason scores, the clinical significance of our result might be limited. 

In the literature, only one study had previously investigated the prognostic values of adipose tissue qualitative features in prostate cancer [24]. In this study, CT-attenuation of SAT was measured at the L4-L5 spine level of 171 patients with high-risk prostate cancer. The results revealed that patients who were in the lowest quartile of SAT HU had significantly lower risk of biochemical recurrence [24]. Similarly, we found that one of qualitative SAT parameters, SAT SUV, was positively associated with N stage and DPFS, suggesting that the qualitative features of SAT may affect the progression of prostate cancer. However, the results of the present study also showed that PPAT SUV was significantly associated with the T stage and M stage as well as N stage, and among all adipose tissue parameters, only PPAT SUV was an independent predictor of survival in the final multivariate model. Moreover, patients with high PPAT SUV were likely to experience disease progression within one year than those with low PPAT (22.2% vs. 3.1%). Therefore, PPAT SUV could be the adipose tissue parameter most closely associated with the clinical course of prostate cancer, and may have a role as an imaging biomarker for reflecting the prostate cancer aggressiveness and prognosis. However, because of the lack of clinical implication and additional clinical value, FDG PET/CT is not routinely performed in the initial staging of prostate cancer [43,44]. Considering that HU and SUV of PPAT showed significant moderate positive correlation with each other and had similar results of correlation analyses with tumor factors and two-year DPFS rates, PPAT HU from diagnostic CT images might be a suitable imaging parameter for assessing the characteristics of PPAT in routine clinical practice of patients with prostate cancer. 

In previous studies regarding PPAT in prostate cancer patients, quantity has been assessed as the PPAT-related imaging parameter [8,11,13,20,45,46,47]. The thickness and volume of PPAT and the ratio of PPAT area to total periprostatic area have been measured using CT and MRI for PPAT quantification, and multiple studies have shown that these quantitative parameters are increased in patients with high Gleason scores and high-risk prostate cancer [13,20,21,47,48]. Furthermore, among patients treated with androgen deprivation therapy, patients with high thickness and volume of PPAT showed significantly worse progression-free survival [44,45]. Conversely, two other studies failed to show any significant relationship between PPAT quantity and prostate cancer aggressiveness [8,11]. Considering that no significant correlation between BMI and either PPAT HU or SUV was observed in our study, these inconsistent results might be due to the confounding effects of qualitative features of PPAT. Recently, an attempt has been made to pharmacologically modify PPAT features with 5α-reductase inhibitors and estrogen, which is expected to affect the outcomes of prostate cancer [49,50]. Based on the results of the present study, PPAT HU and SUV might be used to select candidates and to monitor treatment response to future therapy that targets PPAT in patients with prostate cancer. 

The present study had several limitations that need to be addressed. First, the study was retrospectively performed at a single medical center with a small number of patients and relatively short follow-up duration. Further, because only patients who underwent staging FDG PET/CT due to abnormal findings on other examinations were enrolled, selection bias is inevitable. Therefore, further studies are necessary to validate our results. Second, there is no consensus on how to define PPAT on imaging examinations [10]. Hence, different body levels have been used to delineate PPAT in previous studies, which might affect the results [10]. Lastly, because of the retrospective design of the study, the exact mechanism underlying the association between PPAT parameters and prognosis cannot be explained. Further investigation into the relationship of histopathological findings and the biologic activity of PPAT with its CT-attenuation and SUV is needed. 

## 5. Conclusions

In this study, we measured CT-attenuation and FDG uptake of PPAT from staging FDG PET/CT images in patients with prostate cancer. We demonstrated that FDG uptake of PPAT was independently associated with DPFS after adjusting for tumor stage, serum PSA, and tumor SUV. PPAT showed different qualitative features from SAT and VAT, and in correlation analysis, both PPAT HU and SUV showed significant positive correlations with the T, N, and M stages of prostate cancer and serum PSA level. In survival analysis, patients with high PPAT HU and SUV had significantly worse DPFS than those with low values. The results of our study suggest an imaging evidence of close interaction between prostate cancer cells and PPAT, but further studies are needed to validate our results.

## Figures and Tables

**Table 1:** Baseline characteristics of the patients (*n* = 77).

Characteristics		No. of Patients (%)	Median (Range)
Age (years)			73 (53–89)
Body mass index (kg/m^2^)			24.6 (16.5–41.4)
Gleason grade group	Group 1	13 (16.9%)	
	Group 2	12 (15.6%)	
	Group 3	9 (11.7%)	
	Group 4	19 (24.7%)	
	Group 5	24 (31.2%)	
Serum PSA (ng/mL)			15.5 (1.3–2845.0)
T stage	T2 stage	30 (39.0%)	
	T3 stage	34 (44.2%)	
	T4 stage	13 (16.9%)	
N stage	N0 stage	51 (66.2%)	
	N1 stage	26 (33.8%)	
M stage	M0 stage	55 (71.4%)	
	M1 stage	22 (28.6%)	
TNM stage	Stage II	21 (27.3%)	
	Stage III	20 (26.0%)	
	Stage IV	36 (46.8%)	
Tumor SUV			3.54 (1.72–14.88)
SAT	HU		−100.79 (−119.66–−75.36)
	SUV		0.31 (0.14–0.54)
VAT	HU		−96.75 (−113.51–−73.64)
	SUV		0.46 (0.22–0.80)
PPAT	HU		−84.52 (−104.18–−63.20)
	SUV		0.66 (0.33–1.21)
Treatment	Hormone treatment	35 (45.5%)	
	Radiotherapy	18 (23.4%)	
	Surgery	14 (18.2%)	
	Surgery + hormone treatment	8 (10.4%)	
	Surgery + radiotherapy	1 (1.3%)	
	Radiotherapy + Hormone treatment	1 (1.3%)	

HU, Hounsfield unit; PPAT, periprostatic adipose tissue; PSA, prostate-specific antigen; SAT, subcutaneous adipose tissue; SUV, standardized uptake value; VAT, visceral adipose tissue.

**Table 2:** Relationship of PPAT HU and SUV with Gleason grade group and tumor stage.

Variables		PPAT HU	PPAT SUV
Gleason grade group	Grade 1	−86.63 ± 6.18	0.66 ± 0.17
	Grade 2–3	−86.91 ± 7.47	0.63 ± 0.16
	Grade 4–5	−83.28 ± 8.18	0.67 ± 0.20
	*p*-value *	0.173	0.622
T stage	T2 stage	−86.92 ± 9.06	0.65 ± 0.17
	T3 stage	−85.60 ± 5.27	0.62 ± 0.19
	T4 stage	−80.62 ± 8.96	0.77 ± 0.16
	*p*-value *	0.048 ^‡^	0.022 ^‡^
N stage	N0 stage	−86.98 ± 7.12	0.59 ± 0.15
	N1 stage	−80.61 ± 7.45	0.79 ± 0.17
	*p*-value ^†^	<0.001	<0.001
M stage	M0 stage	−86.51 ± 7.16	0.62 ± 0.17
	M1 stage	−80.64 ± 7.91	0.76 ± 0.17
	*p*-value ^†^	0.007	0.002

All data were expressed as average ± standard deviation, * *p*-values for Kruskal-Wallis test, ^†^ *p*-values for Mann-Whitney U test. ^‡^ On post-hoc analysis, patients with T4 stage disease showed significantly higher values than those with T2 and T3 stage disease (*p* < 0.05), whereas no significant difference was shown between patients with T2 and T3 stage disease (*p* > 0.05). HU, Hounsfield unit; PPAT, periprostatic adipose tissue; SUV, standardized uptake value.

**Table 3:** Univariate analysis for recurrence-free survival.

Variables		*p*-Value	Hazard Ratio (95% CI)
Age (1-year increase)		0.272	1.03 (0.98–1.09)
Body mass index (1.00 kg/m^2^ increase)		0.130	0.89 (0.77–1.03)
Gleason grade group (grade 1 vs.)	Grade 2–3	0.620	1.77 (0.18–17.06)
	Grade 4–5	0.008	4.36 (1.47–12.97)
Serum PSA (1.0 ng/mL increase)		<0.001	1.00 (1.00–1.00)
T stage (T2 stage vs.)	T3 stage	0.105	2.95 (0.80–10.91)
	T4 stage	<0.001	10.42 (2.81–38.66)
N stage (N0 vs. N1 stage)		<0.001	9.57 (3.47–26.41)
M stage (M0 vs. M1 stage)		<0.001	9.26 (3.77–22.77)
TNM stage (stage II vs.)	Stage III	0.534	2.14 (0.19–23.63)
	Stage IV	0.006	16.76 (2.23–126.16)
Tumor SUV (1.00 increase)		<0.001	1.21 (1.09–1.35)
SAT HU (1.00 HU increase)		0.235	1.04 (0.98–1.10)
SAT SUV (0.10 increase)		<0.001	2.77 (1.68–4.57)
VAT HU (1.00 HU increase)		0.457	1.02 (0.97–1.08)
VAT SUV (0.10 increase)		0.006	1.60 (1.15–2.24)
PPAT HU (1.00 HU increase)		0.008	1.09 (1.02–1.16)
PPAT SUV (0.10 increase)		<0.001	1.57 (1.26–1.96)

CI, confidence interval; HU, Hounsfield unit; PPAT, periprostatic adipose tissue; PSA, prostate-specific antigen; SAT, subcutaneous adipose tissue; SUV, standardized uptake value; VAT, visceral adipose tissue.

**Table 4:** Multivariate analysis for recurrence-free survival.

Model	Variables	Model with PPAT HU		Model with PPAT SUV	
		*p*-Value	HR (95% CI)	*p*-Value	HR (95% CI)
Model 1	Age (1-year increase)	0.576	1.02 (0.96–1.08)	0.141	1.04 (0.99–1.11)
	Body mass index(1.00 increase)	0.437	0.94 (0.80–1.10)	0.201	0.90 (0.77–1.06)
	PPAT HU (1.00 increase)	0.021	1.08 (1.01–1.16)	-	-
	PPAT SUV (0.10 increase)	-	-	<0.001	1.72 (1.33–2.21)
Model 2	Gleason grade 2–3(vs. grade 1)	0.525	2.10 (0.21–20.56)	0.549	2.01 (0.20–19.64)
	Gleason grade 4–5(vs. grade 1)	0.329	2.78 (0.36–21.74)	0.212	3.91 (0.46–33.20)
	TNM stage III (vs. stage II)	0.648	1.78 (0.15–20.97)	0.510	2.36 (0.18–30.28)
	TNM stage IV (vs. stage II)	0.036	10.41 (1.16–93.14)	0.017	7.55 (3.01–18.89)
	PPAT HU (1.00 increase)	0.033	1.43 (1.11–1.92)	-	-
	PPAT SUV (0.10 increase)	-	-	0.007	1.50 (1.11–1.94)
Model 3	Serum PSA (1.0 increase)	0.019	1.00 (1.00–1.01)	0.017	1.00 (1.00–1.00)
	Tumor SUV (1.00 increase)	<0.001	1.21 (1.08–1.35)	0.004	1.21 (1.06–1.39)
	PPAT HU (1.00 increase)	0.008	1.09 (1.02–1.15)	-	-
	PPAT SUV (0.10 increase)	-	-	0.001	1.64 (1.29–2.08)
Model 4	SAT SUV (0.10 increase)	0.046	2.13 (1.01–4.47)	0.039	2.15 (1.04–4.47)
	VAT SUV (0.10 increase)	0.705	1.10 (0.68–1.78)	0.759	0.92 (0.56–1.53)
	PPAT HU (1.00 increase)	0.101	1.06 (0.99–1.13)	-	-
	PPAT SUV (0.10 increase)	-	-	0.005	1.45 (1.12–1.87)
Model 5	TNM stage III (vs. stage II)	0.355	3.14 (0.28–35.58)	0.247	4.24 (0.37–48.50)
	TNM stage IV (vs. stage II)	0.032	10.29 (1.22–87.15)	0.013	4.32 (1.12–16.58)
	Serum PSA (1.0 increase)	0.055	1.00 (1.00–1.00)	0.050	1.00 (1.00–1.00)
	Tumor SUV (1.00 increase)	0.273	1.08 (0.94–1.23)	0.054	1.14 (1.00–1.31)
	SAT SUV (0.10 increase)	0.100	1.37 (0.94–2.00)	0.129	1.59 (0.87–2.91)
	PPAT HU (1.00 increase)	0.343	1.03 (0.97–1.10)	-	-
	PPAT SUV (0.10 increase)	-	-	0.003	1.50 (1.15–1.96)

CI, confidence interval; HR, hazard ratio; HU, Hounsfield unit; PPAT, periprostatic adipose tissue; PSA, prostate-specific antigen; SAT, subcutaneous adipose tissue; SUV, standardized uptake value; VAT, visceral adipose tissue.

## Supplementary Materials

The following are available online at https://www.mdpi.com/2075-4426/10/4/185/s1, Supplementary Figure S1: Distribution of computed tomography-attenuation (Hounsfield unit) of periprostatic adipose tissue according to T stage, N stage, and M stage and standardized uptake value of periprostatic adipose tissue according to T stage, N stage, and M stage. Table S1: Relationship of the HU and SUV of the SAT and VAT with Gleason grade group and tumor stage.
