# Supplementary material for: Prognostic Value of CT-Attenuation and 18F-Fluorodeoxyglucose Uptake of Periprostatic Adipose Tissue in Patients with Prostate Cancer
Source: J Pers Med. 2020 Oct 22;10(4):185. doi: 10.3390/jpm10040185 (PMC7711777; doi:10.3390/jpm10040185)
Supplement: Supplementary file 1 [file jpm-10-00185-s001.pdf]

**Supplementary Figure S1.** Distribution of computed tomography-attenuation (Hounsfield unit) of periprostatic adipose tissue according to T stage (a), N stage (b), and M stage (c) and standardized uptake value of periprostatic adipose tissue according to T stage (d), N stage (e), and M stage (f).

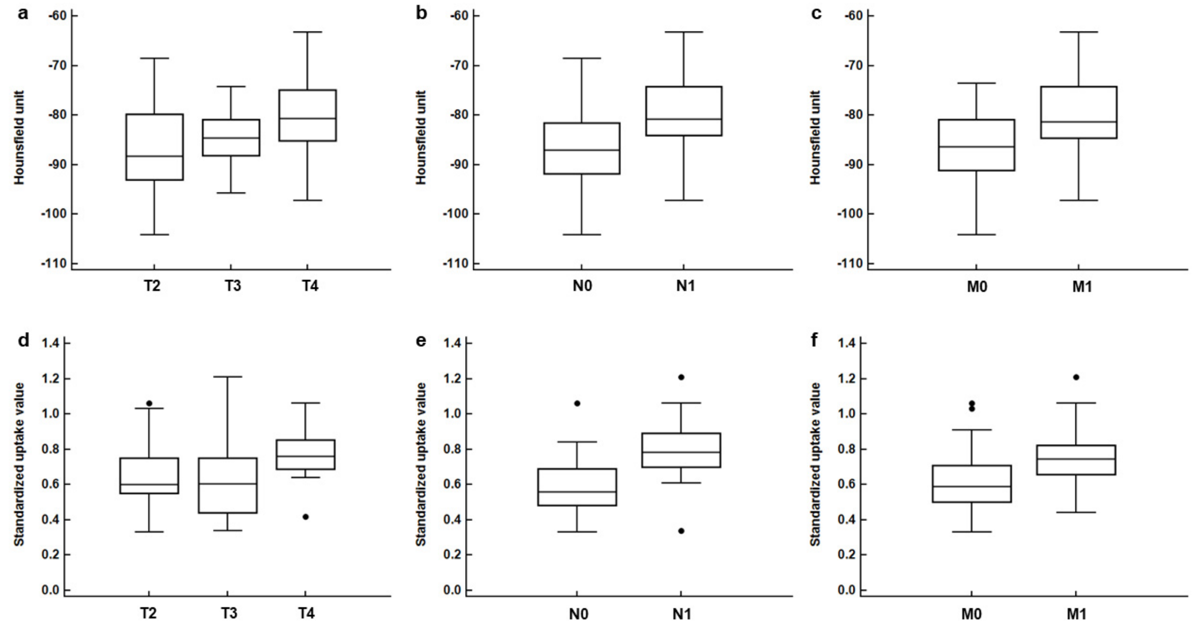


**Supplementary Table S1**. Relationship of the HU and SUV of the SAT and VAT with Gleason grade group and tumor stage.

| Variables | | SAT HU | SAT SUV | VAT HU | VAT SUV |
|---|---|---|---|---|---|
| Gleason grade group | Grade 1 | -101.63±5.92 | 0.30±0.04 | -98.31±8.37 | 0.47±0.08 |
| | Grade 2-3 | -101.72±7.31 | 0.31±0.08 | -99.65±7.70 | 0.48±0.14 |
| | Grade 4-5 | -97.52±8.96 | 0.32±0.09 | -93.16±8.42 | 0.50±0.15 |
| | P-value | 0.047 | 0.879 | 0.012 | 0.890 |
| T stage | T2 stage | -100.18±8.88 | 0.33±0.06 | -98.11±8.99 | 0.51±0.11 |
| | T3 stage | -98.94±6.23 | 0.29±0.09 | -94.70±7.80 | 0.46±0.15 |
| | T4 stage | -98.58±11.51 | 0.35±0.09 | -93.35±9.44 | 0.52±0.14 |
| | P-value | 0.548 | 0.059 | 0.166 | 0.156 |
| N stage | N0 stage | -99.95±7.37 | 0.30±0.07 | -95.91±8.27 | 0.46±0.12 |
| | N1 stage | -98.21±9.85 | 0.35±0.09 | -93.62±9.15 | 0.54±.15 |
| | P-value | 0.457 | 0.006 | 0.198 | 0.058 |
| M stage | M0 stage | -100.11±7.18 | 0.31±0.07 | -97.21±7.96 | 0.47±0.12 |
| | M1 stage | -97.48±10.47 | 0.34±0.10 | -92.28±9.50 | 0.54±0.15 |
| | P-value | 0.223 | 0.179 | 0.046 | 0.072 |

*On post-hoc analysis, patients with grade 4-5 showed significantly higher values than those with grade 1 and grade 2-3 ($p<0.05$), whereas no significant difference was shown between patients with grade 1 and grade 2-3 ($p>0.05$).

HU, Hounsfield units; SAT, subcutaneous adipose tissue; SUV, standardized uptake value; VAT, visceral adipose tissue
